# Malposition of percutaneous endoscopic-guided gastrostomy: Guideline and management

**DOI:** 10.4103/0972-9941.40989

**Published:** 2008

**Authors:** Siamak Milanchi, Matthew T Wilson

**Affiliations:** Department of Surgery, Cedars-Sinai Medical Center, Los Angeles, CA, USA; 1 Trauma Services, Department of Surgery, Cedars-Sinai Medical Center, Los Angeles, CA, USA

**Keywords:** Gastrocolic fistula, gastrocutaneous fistula, percutaneous endoscopic-guided gastrostomy

## Abstract

Percutaneous endoscopic-guided gastrostomy (PEG) is done routinely on patients who suffer from inability to feed by mouth. PEG is generally considered a safe procedure with a low complication rate. A commonly underreported complication of PEG is malposition. This manuscript is a guideline to diagnosis and management of PEG malposition. We describe the different types of malposition, their diagnosis and management.

## INTRODUCTION

**Figure 1:**
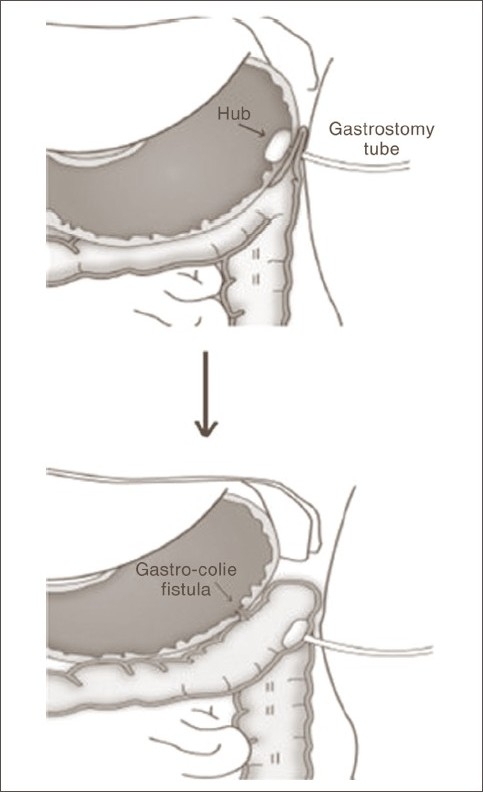
Migration of the malpositioned PEG from stomach into the colon and creation of gastrocolic fistula

Percutaneous Endoscopic-guided Gastrostomy (PEG) is done routinely on patients with inability to feed by mouth. PEG is considered a safe procedure when performed by experienced physicians with endoscopic training. As with any surgical procedure there are a number of known complications. One such complication of PEG placement is malposition. Malposition happens when the gastrostomy tube or its hub is placed in an organ other than the stomach. This includes small bowel, large bowel, peritoneal cavity or abdominal wall. PEG malposition can occur acutely while inserting the tube or as a result of chronicity. PEG placement utilises the Soldinger technique. A large-bore needle is inserted into the anterior abdominal wall after visualising an adequate light reflex of the gastroscope and when minimal palpation of the abdominal wall demonstrates maximal visualised depression of the gastric wall. This needle then enters the stomach under endoscopic guidance. A guidewire is then passed through this needle into the stomach. When inserting the needle into the stomach, if a loop of small or large bowel is between the stomach and the abdominal wall, it may be caught by the needle. As a result, the PEG tube will be inserted through the bowel wall into the stomach. As time passes the tract around the gastrostomy tube epithelialises and forms a gastrocutaneous fistula. This tract is usually well formed and mature in four to six weeks therefore if dislodged the PEG tube can be changed, replaced or removed without complications. Removal or replacement of the tube before this time will cause extravasation of gastric contents and air into the peritoneal cavity. If there is any suspicion about formation of the gastrocutaneous tract a fistulogram using gastrograffin is diagnostic. If the PEG tube is accidentally inserted through the colon into the stomach, this connection will form a gastrocolic fistula [Figure 1].

At the completion of the procedure, PEG should be always secured to the skin so that it is taut. If, however, the PEG is too tight and under constant tension it has the tendency to erode through the anterior gastric wall with eventual migration into the various layers of the abdominal wall. If the PEG is placed through the colon into the stomach, constant tension may cause erosion of the hub through the anterior gastric wall into the colon.

## TYPES OF PEG MALPOSITION

### PEG in the peritoneal cavity

Insertion of the PEG directly into the peritoneal cavity instead of the stomach is very rare since this procedure is done under endoscopic guidance. More commonly, the PEG may later migrate into the peritoneal cavity after eroding through the anterior stomach wall because of constant tension. When this happens early after PEG placement, gastric contents and air enter the peritoneal cavity and cause peritonitis. Subsequent feeding into the peritoneal cavity will cause peritonitis as well. The intraperitoneal PEG is diagnosed with certainty via gastrograffin infusion into the malpositioned PEG which reveals free intra-peritoneal contrast. The intraperitoneal PEG should be removed immediately and the stomach should be repaired. In order to prevent chronic migration of the PEG into the peritoneal cavity it should be secured to the abdominal wall, but not under constant tension.

### Insertion of the PEG through the colon into the stomach

**Figure 2:**
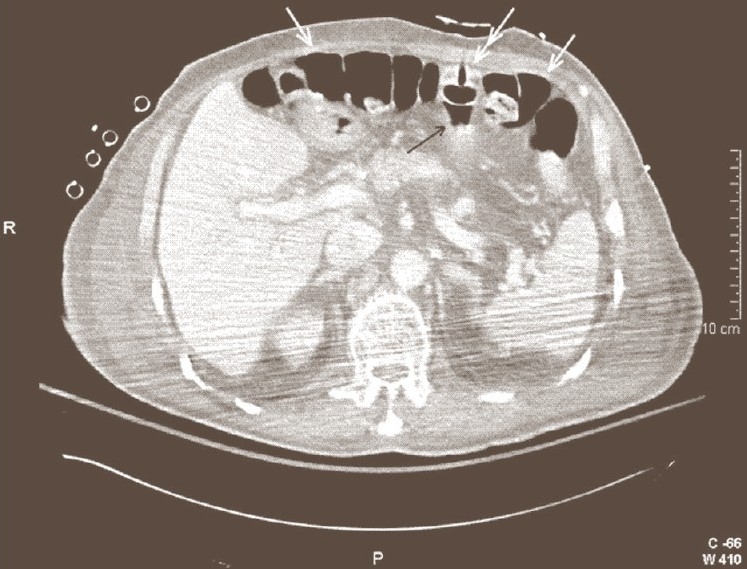
One day after PEG tube placement. The gastrostomy tube (double arrow) has been inserted through the transverse colon (white arrow) into the stomach (black arrow)

**Figure 3:**
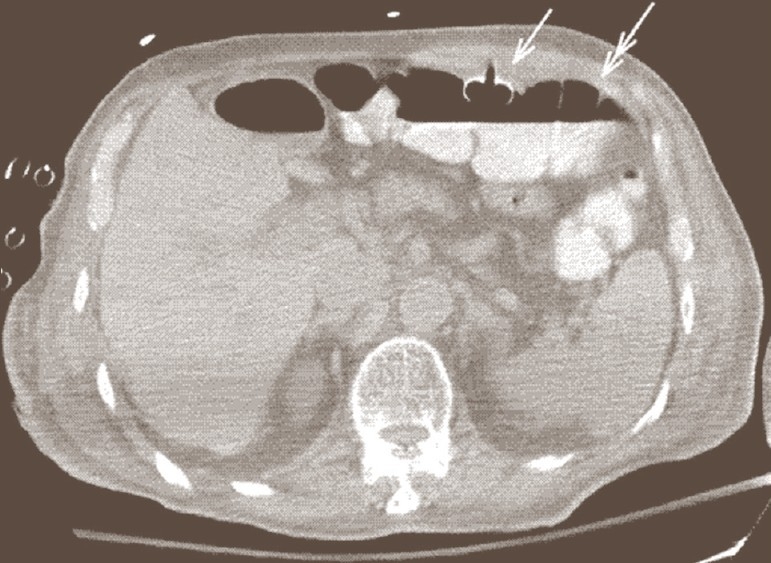
Same patient in Figure 2. Eighteen days after placement of PEG tube (white arrow). Note that the hub of the gastrostomy tube has migrated from the stomach into the transverse colon (double arrow)

In this case, the hub is in the stomach but the tube passes through the colon. This happens if the colon (usually transverse colon) is caught between the stomach and the anterior abdominal wall when inserting the large-bore needle as described earlier. These patients may present early with pneumoperitoneum or peritonitis. Although, pneumoperitoneum has been reported to be benign after PEG placement, early pneumoperitoneum after PEG may be indeed a sign of bowel injury.[1] When detected early, the PEG should be removed and the colonic injury should be repaired. If the PEG is secured to the abdominal wall, it may compress the involved segment of the colon so that the patient will be asymptomatic with no free air [Figures 2 and 3]. If at any time this PEG is dislodged, it will cause spillage from the colon and subsequent peritonitis which needs exploration, removal of the PEG and repair of the stomach and colon. If the PEG migrates from the stomach into the colon the patient may develop severe diarrhoea (secondary to the osmotic load of the formula entering the colon) and halitosis. Gastrocolic and colocutaneous fistulas develop in this type of PEG malposition. Gastrocolic fistula should be considered in any patient with PEG who develops diarrhoea after replacement of the gastrostomy tube.[2] Diarrhoea following the replacement of PEG tube is indicative of gastrocolic fistula.[2] Treatment of gastrocolic fistula is exploration and excision of the fistula and resection of the involved colon segment.[3] Colocutaneous fistula causing severe diarrhoea is reported after replacement of the original well-functioning PEG tube with a Mic-Key button (which is shorter than PEG tube) which subsequently migrated to the colon.[4] PEG malposition through the colon may be also unrecognized for long periods of time. Changing the gastrostomy tube may reveal this problem since the new tube is advanced into the colon, instead of stomach.[5] Once the gastrostomy tube is removed the colocutaneous fistula will collapse and close, otherwise it can be resected. Placement of PEG through the colon may also cause obstruction of the colon which needs exploration, removal of the gastrostomy tube and repair of the stomach and colon.

Occasionally alternative and non-traditional methods are used to manage PEG malposition. The authors of this manuscript recently reviewed a case of malpositioned PEG in the colon detected 4 weeks after placement. This was treated by cutting the gastrostomy tube at the skin and then pushing the rest of the tube into the colon which was later found in the stool. In this case, the gastrocolic fistula was treated by endoscopic clipping of the gastric opening of the fistula. There are not enough data in the literature to support these non-standard approaches to PEG malposition. The outcomes are not standardised and are based on the experience and discretion of the surgeon.

### Insertion of the PEG through the small bowel into the stomach

This is usually detected soon after the procedure since it causes early pneumoperitoneum, peritonitis or small bowel obstruction. It is unlikely that this type of malposition remains undetected and asymptomatic. Treatment of this type of PEG malposition is removal of the gastrostomy tube and repair of the small bowel.

### Buried bumper syndrome (BBS)

A tight PEG may erode through the anterior gastric wall and be embedded in the abdominal wall. This is usually a late complication of PEG placement and may cause gastrointestinal bleeding, perforation, peritonitis and even death.[6] Soft tissue infection of the abdominal wall with or without abscess is described as well. Infection of the abdominal wall requires incision, drainage and antibiotics. Treatment of BBS is removal of the PEG and emergency repair of the stomach. If BBS happens late it usually won't cause peritonitis since the gastrocutaneous fistula is already formed and there should be no free spillage of the gastric contents into the peritoneal cavity. In this case the PEG needs to be removed and replaced immediately by a gastrostomy tube (or foley catheter) before the tract collapses.

### Accidental/inadvertent removal of the PEG

This condition is not uncommon in patients with altered mental status secondary to neurosurgical injuries or in chronically ill patients, dependant on nursing care. If this happens early after PEG placement patients develop peritonitis. The PEG should be removed and the stomach should be repaired. Nonoperative management of the early dislodged PEG tube has been described in the literature (nasogastric suctioning, intravenous antibiotics, observation) followed by delay placement of a new tube, although there is not enough data to justify this method.[7]

If a patient still needs gastrostomy tube, the early dislodged PEG should be replaced by laparoscopy or laparotomy and any blind attempt to replace the PEG should be avoided.[8] Late spillage of the gastric contents into the peritoneal cavity is less likely since a gastrocutaneous fistula is already formed. In this case the PEG should be replaced through the old gastrostomy site immediately before the tract collapses.

### Loose PEG

Although not a malposition, a loose PEG can have serious complications. After PEG placement the gastrostomy tube is secured and lightly tightened to the skin therefore the stomach is secured to the abdominal wall. Gastrostomy tube should be kept taut at the skin for at least four to six weeks. If the gastrostomy tube becomes loose before this time, gastric contents and air will enter the peritoneal cavity and cause peritonitis. Treatment of a loose PEG is pulling and securing the tube to the skin. If the patient already has peritonitis then laparotomy with irrigation of the peritoneal cavity should be done. If the patient has mild tenderness around the PEG site and is not septic then a trial of intravenous antibiotics and NPO with gastric suctioning can be done, in addition to securing the gastrostomy tube to the skin.

## DIAGNOSTIC IMAGING OF PEG MALPOSITION

1. It can be difficult to diagnose colon injury following PEG placement[9] although presence of free air and free flluid and extravasation of fluid into the peritoneal cavity in the CT scan are clues to this diagnosis. CT scan with oral gastrograffin (or contrast injected in the PEG tube) is the imaging method of choice for PEG malposition. CT scan shows the position of the hub and the tube.
2. Contrast study of the gastrostomy tube by injecting gastrograffin in the tube and taking plain abdominal X-ray or doing fluoroscopy will show the position of the hub and any extravasation of the contrast into the peritoneal cavity.
3. Endoscopy of the stomach may show absence of PEG in the stomach which confirms its migration from the stomach. Gastrocolic fistula or a scar at the gastrostomy site may be seen as well. Colonoscopy verifies position of the hub in the colon.[10]
4. Gastrograffin enema identifying a filling defect in the colon can verify the position of the hub in the colon.[10]

## CONCLUSION

PEG malposition can have disastrous results. Complications can occur early or late after PEG placement. Early recognition and treatment of this complication is crucial. Clinicians should be aware of symptoms and signs of PEG malposition and use appropriate diagnostic studies. On time and safe treatment can solve this problem.
